# Sociality and self-awareness in animals

**DOI:** 10.3389/fpsyg.2022.1065638

**Published:** 2023-01-09

**Authors:** Yanyu Lei

**Affiliations:** Independent Researcher, Winnipeg, MB, Canada

**Keywords:** self-awareness, self-recognition, mirror test, sociality, animal cognition

## Abstract

Recognizing one’s mirror reflection appears to be a simple task, but beyond humans, few animals have demonstrated this capability. Mirror self-recognition is indicative of self-awareness, which is one’s capacity for self-directed knowledge. This theoretical paper examines literature from the past 50 years regarding self-recognition in over 30 species. Animals are classified based on the quantity and quality of research supporting evidence of their self-recognition abilities. Additionally, animals are classified as either social or solitary. It was found that only social animals have consistently demonstrated self-recognition, while solitary species studied so far do not seem to possess this trait. This finding aligns with the social intelligence hypothesis. This paper also reveals a lack of research on solitary species and recommends future studies examine self-recognition in these animals. A meta-analysis quantifying sociality on a numerical scale is also recommended. Given the existing evidence, this article proposes that social animals are more likely to be self-aware than solitary species.

## Introduction

Self-awareness is among the most mysterious of cognitive capabilities. Self-awareness has been described as “arguably the most fundamental issue in psychology, from both a developmental and evolutionary perspective” (Rochat, 2003, p. 1). Part of the complexity is the lack of consensus on the definition of the term “self-awareness” (Gallagher, 2011). In this article, self-awareness refers to one’s capacity for self-directed attention (Gallup, 1970) and includes knowledge of one’s private mental states such as thoughts and emotions (Morin, 2011). The mirror self-recognition (MSR) test, developed by Gallup (1970) is the main technique used to detect animal self-awareness. In the MSR test, animals typically pass through four stages: (i) social behavior directed at the mirror, followed by (ii) close mirror inspection, (iii) a decline in social behavior, and an increase in mirror-inspection, and finally (iv) self-directed behavior (Plotnik et al., 2006). In the final stage, an animal is anesthetized, and then an odorless mark is placed on a body part that cannot be seen normally. Afterward, a mirror is placed in front of the animal, and if the animal investigates the mark using its reflection, it is regarded as evidence of self-recognition. It is important to distinguish the difference between self-recognition and self-awareness. Mirror self-recognition only represents basic and not full-blown self-awareness. This is because MSR likely only requires kinesthetic self-knowledge and does not necessarily include the level of knowing one’s mental states (Morin, 2011). Thus, an animal could be aware of its own thoughts, but does not show it visually through touching a mark on itself after seeing its mirror reflection. The mirror test would be unable to find self-awareness in this case. Additionally, vision is not the primary sense of all animals, so a sight based MSR test may be unable to detect self-recognition in certain species. For species that primarily use olfactory cues, an animal could recognize its own smell but not its image. Olfactory based self-recognition tests have been conducted, but the visual MSR test remains the most widely used means of testing for self-recognition. However, an inability to pass MSR does not necessarily indicate a lack of self-recognition. This paper reviews existing data on animals tested for MSR and attempts to find a shared characteristic among the animals that have demonstrated at least basic self-awareness through mirror self-recognition, with the goal of shining light on a critical trait associated with self-awareness. Excluding humans (*Homo sapiens*), chimpanzees (*Pan troglodytes*) and orangutans (*Pongo pygmaeus*) have conclusively demonstrated MSR based on consistent, reproducible experimental evidence that has been confirmed by numerous independent studies (Gallup, 1970; Lethmate and Dücker, 1973; Suarez and Gallup, 1981; Hanazuka et al., 2018; Gallup and Anderson, 2019). While only the three great apes are convincingly self-aware, numerous other animals show strongly suggestive signs of self-awareness, as indicated by them passing MSR tests in at least two separate studies without prior training. These species are the bottlenose dolphins (*Tursiops truncatus*; Marten and Psarakos, 1994; Morrison and Reiss, 2018), bonobos (*Pan paniscus*; Westergaard and Hyatt, 1994; Walraven et al., 1995), and, most recently discovered, the cleaner wrasse (*Labroides dimidiatus*; Kohda et al., 2019, 2022). The above animals are all highly social, except for adult male orangutans. Social animals are animals that tend to associate with conspecifics in groups of two or more individuals (American Psychological Association, 2022). Animals with minimal sociality are solitary, meaning they do not associate with other members of their species unless for courtship and mating (Cavalcanti and Gese, 2009). There are some social animals for which the presence of self-awareness is suspected but uncertain. These species either show signs of self-recognition but only had one study to support the findings or the studies suffered controversial results. Examples are orcas (*Orcinus orca*; Delfour and Marten, 2001), Eurasian magpies (*Pica pica*; Prior et al., 2008), garter snakes (*Thamnophis sirtalis*; Burghardt et al., 2021), domestic dogs (*Canis familiaris*; Horowitz, 2017), Asian elephants (*Elephas maximus*; Plotnik et al., 2006), three species of ants: *Myrmica sabuleti, Myrmica rubra, Myrmica ruginodis* (Cammaerts and Cammaerts, 2015), western gorillas (*Gorilla gorilla*; Allen, 2007; Posada and Colell, 2007), pigeons (*Columba livia*; Epstei et al., 1981; Uchino and Watanabe, 2014), the rhesus macaque (*Macaca mulatta*; Chang et al., 2015, 2017; Huttunen et al., 2017), Indian house crows (*Corvus splendens*; Buniyaadi et al., 2020), and Clark’s nutcracker (*Nucifraga columbiana*; Clary and Kelly, 2016). Although few completely solitary animals have been tested for MSR, the ones that have include pandas (*Ailuropoda melanoleuca*), sun bears (*Helarctos malayanus*), and octopuses (*Octopus vulgaris*), all of which have failed to pass the MSR test. Most species that consistently pass or have shown the potential of passing the MSR test share one common trait—high sociality. This indicates a possible association between social animals and self-awareness. This aligns with the social intelligence hypothesis, which suggests that, to deal with complex social environments such as collaboration and relationships, social animals evolved larger brains and greater cognitively capability (Holekamp, 2007). Social species are therefore more likely to be self-aware than solitary ones because their brains are enlarged and more capable of supporting the cognitive capabilities necessary for self-awareness. However, additional research is needed for solitary species given the lack of information on their capacity for self-recognition. This paper investigates many of the species tested for self-recognition, providing a holistic perspective on our current understanding of animal self-awareness. Based on detailed inquiry, this study builds on current theory by proposing the hypothesis that social animals may be more likely to be self-aware as compared with solitary animals.

## Species conclusively showing self-awareness are social

In humans, self-recognition (which is representative of a basic form of self-awareness but does not necessarily include knowledge of one’s own mental states) can arise in some infants at around 15 months of age, when they successfully pass the MSR test by interacting with their mirror reflection by touching a mark on their body that cannot be seen without a mirror. The majority of infants display this capability at 2 years of age (Anderson, 1984). Healthy adults are not only capable of the basic self-awareness that can be concluded *via* passing the mirror test but also full-blown self-awareness that includes understanding of one’s emotions and thoughts. In fact, people experience full self-awareness daily when we recognize ourselves in the mirror and when we manage our emotions. Among non-human animals, chimpanzees demonstrate the most convincing evidence of self-awareness (Gallup, 1970; Lethmate and Dücker, 1973; Suarez and Gallup, 1981; Marino et al., 1994; Gallup and Anderson, 2019), with roughly 75% of young adult chimpanzees passing the MSR test (Robert, 1986). Furthermore, Calhoun and Thompson (1988) found two young chimpanzees capable of retaining self-recognition after 1 year without access to mirrors. Both humans and chimpanzees are extremely social, with chimpanzees capable of forming complex social structures with groups of up to 150 individuals (Goodall, 1986).

Orangutans also exhibit strong evidence for self-awareness by consistently passing the MSR test (Lethmate and Dücker, 1973; Suarez and Gallup, 1981). At first, this may appear contradictory to the hypothesis that social animals are more likely to be self-aware than solitary species because orangutans are solitary. However, recent research shows that young and female orangutans are very social while it is the adult males that are relatively solitary. Mother orangutans and their offspring can remain in continuous contact for up to 7 years and young orangutans frequently socialize with adults and other juveniles (van Noordwijk et al., 2013). Upon reaching maturity, female orangutans typically range near their mothers and form loose communities. In a group of 12 orangutans, adolescent and subadult males showed the most play. But as male orangutans aged, they became less social and took on a largely solitary lifestyle (Poole, 1987). However, even adult males occasionally join large travel bands which form around areas of high fruit abundance (Delgado Jr and Van Schaik, 2000; Kopp and Liebal, 2018). Despite the solitary lifestyle of male orangutans, the species is semi-social overall. Regarding the possible influence of sociality on the development of self-recognition in males, they spend the first several years of their life living with their mothers and peers and are likely to have already developed self-awareness before moving into a solitary lifestyle. Morrison and Reiss (2018) have shown that self-awareness in the other self-aware apes, chimpanzees and humans, can develop at young ages of 2.5 and 2 years, respectively. This early onset of self-awareness is significant when coupled with the fact that it can be retained. Chimpanzees, for example, were able to maintain their level of self-awareness even after 1 year without access to mirrors (Calhoun and Thompson, 1988). The fact that self-awareness develops at a young age and can be retained for a significant timespan means that the solitary lifestyle of a fully-grown male orangutan likely does not affect its capacity for self-awareness as it—like other great apes—already developed self-awareness in its childhood and is simply retaining it in its adult life. Future research studying the onset of self-recognition in orangutans and their ability to retain it are needed to further support this explanation.

## Strongly suggestive signs of self-awareness in social animals

Another great ape, the bonobo, demonstrated self-directed behavior in a study by Walraven et al. (1995) in which four out of seven individuals displayed self-directed behavior. An earlier study involved four out of nine bonobos showing self-directed behavior after being exposed to their mirror reflections (Westergaard and Hyatt, 1994). However, a more recent study could not conclusively support these results. (Shorland et al., 2020). Like the other great apes, bonobos are a gregarious species as evidenced by occasional peaceful ranges involving two different bonobo groups (Furuichi, 2011).

In aquatic species, multiple studies indicate that bottlenose dolphins have passed the MSR test (Marten and Psarakos, 1994; Reiss and Marino, 2001; Herman, 2010). One paper even found that dolphins exhibit self-awareness at an earlier age than humans and chimpanzees (Morrison and Reiss, 2018). However, Reiss and Marino (2001) study lacked an important control condition in which marks are applied to body parts that can be seen without the aid of a mirror. This would allow comparisons to be made with the dolphins’ behavior toward marks that can only be seen with a mirror. If there was no difference in their behavior, then the dolphins may simply be interested in observing the mark itself, thus offering no conclusion of self-recognition. Moreover, all studies involving MSR in aquatic animals are challenged by the animals’ lack of arms, making it difficult for them to show mirror-guided self-directed behavior by physically touching the mark. In MSR tests, dolphins will open their mouths and posture, but these are also social behaviors which makes it difficult to identify true self-directed behavior (Marten and Psarakos, 1995). Thus, these studies are highly suggestive but not a definitive indicator of self-awareness in dolphins. Regarding sociality, dolphins live and hunt in cooperative pods, with males engaging in 2–3 levels of alliance affiliation (Connor, 2007). These behaviors make dolphins one of the most social species in the world.

Recently, two studies suggested that cleaner wrasse have shown convincing signs of self-awareness (Kohda et al., 2019, 2022). Using the MSR test, a colored mark was applied on the fish while they were under anesthesia, done so *via* a process known to not affect their behavior. These marks were applied in places that cannot be seen without using a mirror, such as the throat in the case of four of the fish. The paper’s analysis was restricted to observations of the throat-marked fish because the other marked fish displayed ambiguous behavior that could not be distinguished from natural or mirror-prompted behavior. Upon mirror exposure, three of the four throat-marked fish scraped their throats against the substrate, demonstrating apparent understanding that the reflection is of themselves. The researchers would later conclude that the behavioral response of these cleaner wrasse was consistent with that of other animals which have passed the mirror test (Kohda et al., 2019). While the evidence presented strongly suggests self-recognition in cleaner wrasse, it should be noted that the marks applied on them were brown-pigmented rubbery material, which resembles the ectoparasites they are evolved to remove. It is therefore natural that they would investigate the marks once they view it in the mirror. However, like other species with possible self-awareness, the cleaner wrasse is very social. Cleaning stations, where other fish can go to be cleaned by the cleaner wrasse, are typically composed of either a pair of adults, a group of juveniles, or a group of females and one dominant male (Dunkley et al., 2020). As such, the cleaner wrasse frequently interacts with conspecifics.

The above studies strongly suggest self-awareness in bonobos, dolphins, and the bluestreak cleaner wrasse, all of which are social animals. If these species are indeed self-aware, it would support the proposed hypothesis.

## Uncertainty in some social animals tested for MSR

Some social animals exhibit uncertain signs of self-recognition. For example, orcas show similar results as bottlenose dolphins when tested for self-recognition (Delfour and Marten, 2001) but the conclusions remain challenged for similar reasons (i.e., lack of arms to directly touch the marks). Furthermore, only one study supports the findings in orcas whereas there are multiple for bottlenose dolphins. Thus, despite being a highly social species, the findings are uncertain.

In Asian elephants, one study found two elephants incapable of MSR but capable of using a mirror to find food in the background (Povinelli, 1989). Another study showed one elephant passing the mark test by touching the mark applied to it while the other two elephants showed self-directed behavior but did not touch the mark directly (Plotnik et al., 2006). Thus, only the findings in a single elephant support MSR in the species, making it very uncertain. Asian elephants generally possess complex sociality in the wild, but males are significantly less social than females, which are commonly found in family units (Seltmann et al., 2019). Long-term all-male groups have been found in non-forested areas, but these groups are in looser arrangements than female groups. In forested areas, males remain solitary or in mixed-sex groups (Srinivasaiah et al., 2019).

Ants are an incredibly social group of insects and have displayed impressive teamwork abilities, such as relocating their entire colony. One study found that three species, *Myrmica rubra*, *Myrmica ruginodis*, and *Myrmica sabuleti* have shown potential for self-recognition (Cammaerts and Cammaerts, 2015). When exposed to a mirror, ants of all three species marked with a blue dot would attempt to clean themselves by touching the mark. Similar results were not exhibited when ants were marked with a brown dot, which is the same color as their body. It appears that the ants used their mirror reflection to see the unusual blue dot and attempt to clean it. If true, this behavior would indicate self-recognition. Additional studies are needed to verify these findings.

The presence of self-awareness is perhaps most controversial in western gorillas, which is another social ape (Robbins et al., 2004). Four studies reported a lack of MSR (Suarez and Gallup, 1981; Ledbetter and Basen, 1982; Nicholson and Gould, 1995; Shillito et al., 1999) and two studies showed self-recognition (Allen, 2007; Posada and Colell, 2007). It should be noted that, in the case of the latter two studies, the gorillas had undergone training that made them accustomed to the mirror and familiar with human contact. Unlike other social animals that consistently pass the MSR test, gorillas must be trained to succeed. Similar to gorillas, two studies show that pigeons are capable of locating a mark on their body after extensive training (Epstei et al., 1981; Uchino and Watanabe, 2014). However, it is worth noting that untrained pigeons have never managed to pass the mirror test (de Waal, 2008).

The rhesus macaque was originally thought to be incapable of self-awareness, along with several other species of monkeys (Gallup, 1970). However, newer research indicates the contrary but only after significant training. In two studies, researchers trained the monkeys by shining an irritant laser light on their face in front of a mirror, prompting the monkey to touch the spot. After 2–5 weeks, the monkeys immediately touched their face after seeing a non-irritating red spot in the mirror (Chang et al., 2015, 2017).

Another case of possible self-awareness is found in the Eurasian magpie. When marked with a bright yellow color and presented with a mirror, the magpies immediately exhibited self-directed behavior by attempting to touch the mark with their beaks. Unmarked birds exposed in front of a mirror acted aggressively before calming down soon after (Prior et al., 2008). However, magpies were again analyzed in Soler et al.’s (2020) study but did not show evidence for self-recognition.

Indian house crows have also been tested for self-recognition (Buniyaadi et al., 2020). In the study, four of the six crows with a colored mark attempted to remove it after exposure to a mirror. This was not found in the control group. House crows roost communally and can number up to 3,000 individuals. However, because this study has not been reconfirmed by other independent tests, and because only six individuals were tested, its findings remain uncertain.

Clark’s nutcracker was found to have self-recognition in one study (Clary and Kelly, 2016). The study employs a novel mirror-recognition task using regular and blurry mirrors. Blurry mirrors prevent recognition of small details in identity while retaining general information about contingent motion. Knowledge of contingent motion is gained when one’s own movements correspond with that of the reflection. In a natural environment, it is rare for a bird to receive exposure to small identity details as that would require a clear reflection. With the mark test, the nutcrackers demonstrated greater self-recognition with a blurred reflection. The study explains that the identity information provided *via* a clear reflection could interfere with the contingent motion information because it is less familiar, thus making it harder to self-recognize in a clear mirror than in a blurry one. Nutcrackers are significantly less social compared to other corvids such as pinyon jays (*Gymnorhinus cyanocephalus*) but are not solitary. Pairs form during breeding season, and they defend nests against other conspecifics. Small family groups have been found and, in rare instances, large flocks form when nutcrackers move to lower elevations (Templeton et al., 1999). The adapted mirror recognition test used for the nutcracker is an interesting advancement in assessing self-recognition, but its findings of self-recognition are yet to be confirmed by subsequent studies.

One of the more interesting cases of potential self-awareness is found in the common garter snake, which was tested using a chemically based self-recognition test. A study by Burghardt et al. (2021) tested 24 individually housed garter snakes and found that males differentiated between their own stimuli and that of littermates fed the same diet. The rate of each snake’s tongue-flicking and overall activity around the cage was measured. Snakes flicked their tongues less when presented substrate from a littermate than they did with their own substrate. However, females did not discriminate their own chemical deposits from those left by males eating the same diet while other males showed differentiation. This does not necessarily indicate a lack of self-recognition in female garter snakes as the authors note that this could relate to more intense male–male competition. Garter snakes are highly social reptiles, with the eastern garter snake subspecies showing signs of “friendship” by associating with specific non-random individuals (Skinner and Miller, 2020).

Domestic dogs are highly social canids and frequently interact with both humans and other dogs (Marshall-Pescini and Kaminski, 2014). Dogs use olfaction as their primary sense, rely on olfactory cues for communication and, despite failing the visual MSR, have demonstrated the ability to distinguish their odors in an “olfactory mirror” (Horowitz, 2017). In this study, dogs were presented their own odors with or without another added odor. They spent more time investigating the latter. Dogs also spend more time smelling the odor of other dogs than their own. However, in both dogs and garter snakes, the distinguishing factor comes from the duration of time for which subjects differentiate odors. This is not as clear an indicator of self-recognition as directly touching oneself which is the case with passing the visual MSR. Additional studies are needed.

Although the social animals mentioned in this section are not confirmed to have self-recognition, their inconsistent behaviors do suggest a possible degree of MSR. If, however, these animals are indeed self-aware, then the hypothesis is further supported by an increased number of social species capable of MSR in contrast with solitary ones. This highlights the need for further research to confirm existing findings in these species.

## Completely solitary animals fail the mirror test

Studies have found that three completely solitary animals—animals that only interact for courtship and mating—tested for MSR have failed, supporting the notion that social animals are more likely to be self-aware.

The first of these solitary species is the Malayan sun bear. When tested for MSR, sun bears only spent 13% of their time engaged in aggressive social behavior (Hafandi et al., 2018) toward the mirror, which is stage (*i*) of passing the MSR test (i.e., social behavior directed at the mirror). The 2018 study then concluded that the sun bear appears incapable of self-recognition as it failed to pass the other three stages of the test. Regarding social behaviors, the Malayan sun bear is completely solitary except for occasional mother-cub duos and rare congregations at large fruit trees (Hafandi et al., 2018; The International Association for Bear Research and Management, 2022).

Another solitary species tested for MSR is the octopus, which has shown cannibalistic behavior (Hernandez-Urcera et al., 2014) and even maintains maximum distance while mating (Wells and Wells, 1972). The octopus is regarded as the most intelligent invertebrate (Linden, 2003), thus making it a good subject choice for advanced cognitive tests such as MSR. When octopuses were presented with a mirror, they experienced increased activity levels, but did not exhibit signs of self-awareness (Mather and Anderson, 2007). Another study noted that octopuses did not view their mirror reflections and conspecifics differently (Mather and Kuba, 2018), thus failing the MSR. However, it should be noted that octopuses also depend on chemical and touch senses in addition to vision. When choosing its food, chemicals cues are used more than visual ones (Maselli et al., 2020). To prevent its tentacles from getting tangled, octopus skin produces a chemical that is recognized by its suckers (Moskvitch, 2014). Thus, concluding a lack of self-awareness based on their performance in a visual test may not be fully reliable. Further self-recognition tests, preferably modified to better suit octopuses, are suggested.

Giant pandas are the third solitary species to fail the mirror test. In captivity, where they are close to conspecifics, pandas only spend 1% of their time socializing (Mainka and Zhang, 1994). In nature, both male and female pandas live solitarily (unless the female is caring for a young), and breeding interactions are short and rare (Kleiman and Seidensticker, 1985). One study subjected 34 captive male and female pandas of various ages to MSR tests. The pandas all spent similar amounts of time interacting socially with the mirror, thus passing the first stage of the MSR test. Furthermore, all individuals continuously exhibited aggressive behavior towards their mirror reflection but failed to move on to the third and fourth stages, indicating that they do not recognize their self-image (Ma et al., 2015).

## Failure in some social animals tested for MSR

There are social animals that have failed the MSR test. Sea lions (*Zalophus californianus*), despite boasting complex mental concepts and social behavior, have failed (Delfour and Marten, 2001). The New Caledonian crow (*Corvus moneduloides*; Medina et al., 2011), common hill myna (*Gracula religiosa*; Lin et al., 2021), African gray parrot (*Psittacus erithacus*), Jungle crow (*Corvus macrorhynchos*; Kusayama et al., 2000), and the great tit (*Parus major*; Kraft et al., 2017) all fail to show MSR. The jackdaw (*Corvus monedula*; Soler et al., 2014) and Californian scrub-jay (*Aphelocoma californica*; Clary et al., 2020) demonstrated interest in the mirror, but could not pass the mark test. The authors of both studies highlight the need for alternative approaches in measuring avian self-recognition. In primates, the cotton-top tamarin (Hauser et al., 2001) also did not show MSR. These studies indicate that social behavior is not the sole factor determining self-awareness. However, the possible influence of social behavior on self-recognition should not be neglected. Presently, no completely solitary species has shown any sign of self-recognition (Table 1).

**Table 1:** Summary of social and solitary animals by their self-awareness.

Self-awareness	Social/semi-social	Solitary
Conclusively self-aware	Humans, chimpanzees, orangutans	Unknown
Very possibly self-aware	Bonobos, dolphins, cleaner wrasse	Unknown
Uncertain self-awareness	Orcas, Eurasian magpies, Elephants, three species of ants (*M. sabuleti, M. rubra, M. ruginodis*), pigeons, western gorillas, rhesus macaques, garter snakes, domestic dogs, Indian crows, Clark’s nutcracker	Unknown
Not self-aware	Sea lions, New Caledonian crow, common hill myna, African gray parrot, cotton-top tamarin, jungle crow, Californian scrub-jay, great tit, jackdaw	Pandas, octopuses, sun bears

## Why social animals are more likely to be self-aware

Self-awareness is a cognitive capability possessed by animals with advanced cognition. Social animals are more likely to possess more complex cognitive abilities—and therefore self-awareness—because of the widely supported social intelligence hypothesis (SIH). The SIH posits that humans and other social animals evolved larger brains and more sophisticated cognitive abilities in response to challenges brought by complex social environments (Johnson-Ulrich, 2018). Humphrey’s (1976) study suggests that social interactions such as collaboration, disagreement, family relations, and friendship offer more cognitive challenges than physical problems such as independently hunting for food. Social animals are hence exposed to more opportunities for cognitive development than solitary ones which interact more frequently with less demanding physical challenges. As a species becomes more cognitively advanced due to their social environment, so will their ability to support more complex societies, which in-turn enhances their cognitive capabilities, making for the co-evolution of cognitive and social complexity. This provides social species greater cognitive capabilities and therefore a higher probability of developing self-awareness. Additionally, socialization may stimulate cognitive abilities necessary for self-awareness but not directly tied to intelligence. This would explain why some highly intelligent solitary animals such as the octopus fail the MSR test, because they lack certain cognitive capacities that are related to high sociality but not necessarily related with high intelligence. What these cognitive capacities are specifically require further research.

While substantial supporting evidence of the SIH is found in primates, research has confirmed its predictions in numerous other animals. Sakai et al.’s (2011) study compared the brain size of four hyena species of differing social level. It found that the spotted hyena, the most social of the four, had the largest brain volume relative to body size which is consistent with the SIH’s predictions. In elephants, the SIH gains strong support as they possess some of the largest mammal brains and are extremely social. Findings in cetaceans show pod size and relative brain size are associated, which is consistent with the SIH (Marino, 2002). Since the SIH is supported by findings from such a wide range of social species, its implications on the development of cognitive traits such as self-awareness may also be relevant for many social species. This provides the explanatory foundation for why social animals are more likely to be self-aware.

## Discussion

Currently, there is significant data on self-awareness in social animals, but little information on solitary species (Ma et al., 2015). Although all animals that have passed the MSR are all social, sociality does not automatically suggest that animals are self-aware. Some social animals fail to demonstrate self-recognition. To confirm any hypothesis that compares social and solitary animals and their capacity for self-awareness, additional research must be conducted on MSR in solitary species. This paper recommends monitor lizards (Varanidae) for future MSR testing because they are highly solitary but also very intelligent (Pianka and King, 2004; Northcutt, 2013; Güntürkün et al., 2020; Howard and Freeman, 2022). In particular, the Komodo dragon (*Varanus komodoensis*) is an attractive candidate. They live solitarily and only occasionally congregate at large carcasses (MacLean, 1978). Less is known about the cognitive ability of Komodo dragons, so a self-recognition test would also shed light on this aspect of study.

The hypothesis presented in this paper also serves as preliminary support for a more comprehensive meta-analysis. A future study quantifying sociality on an ordinal or numeric scale instead of a binary social versus non-social categorization is highly recommended. This helps mitigate the lack of research in solitary species as using graded sociality allows for more precise comparisons within social species (for which information on self-recognition is abundant). For example, it is possible that a highly social animal like the Indian house crow is more capable of self-recognition than a semi-social animal like Clark’s nutcracker. This paper recommends starting the meta-analysis with corvids because the social behavior of species within the same family is more comparable. Corvids display varying levels of sociality and numerous corvid species have already been tested for self-recognition. Subsequent studies can quantify variables such as group size and association level for drastically different species, expanding toward a comprehensive scale including all the animals covered in this study.

Many uncertainties remain in the study of animal self-awareness. The mark test itself remains a debatable measure of self-awareness, with the major question: does failing the MSR test mean a lack of self-awareness? It is possible that some self-aware animals simply do not care about the mark but do possess awareness of their private mental thoughts and an understanding of self. As shown in tests with garter snakes, dogs, and octopuses, the MSR test is subject to sensory limitations, creating a possible bias against animals that do not use vision as their primary sense. Failing the visual MSR does not necessarily mean the species cannot self-recognize. Additionally, as is the case with the gorillas and rhesus macaques, it is unknown if trained self-directed behavior necessarily means natural self-recognition. Moreover, Gallup and Anderson’s (2019) review concludes that differences in self-awareness in different animals may be qualitative rather than quantitative. Thus, developing more objective and reliable self-recognition tests, perhaps designed individually for one species or a group of similar species, remains a future challenge.

Another area of future research is whether self-recognizing, non-human animals can know their internal mental states such as emotions. A basic level of self-awareness is confirmed to be present in non-human animals that conclusively pass the mirror test, but it is unknown if this can be extended to include their knowledge of internal thoughts and emotions.

A basic level of self-knowledge is guaranteed in animals that pass the mirror test, but it is unknown if they possess advanced understanding of private mental states. Despite uncertainties in the methodology of determining self-awareness and a scarcity of information on solitary species, the existing data appears firm in the pattern of social animals being more likely to be self-aware than non-social ones because no solitary species has shown self-recognition. Among the species analyzed in this article, the conclusively self-aware animals were social to some degree, with humans and chimpanzees being highly social and orangutans being semi-social. Other animals exhibiting strongly suggestive signs of self-awareness were also highly social. These are bonobos, bottlenose dolphins, and the bluestreak cleaner wrasse. Certain species showed signs of MSR in individual studies or after training, but the research lacks independent verification by additional studies to be considered strongly suggestive of self-awareness. Examples include the orca, Eurasian magpie, Asian elephant, ant, western gorilla, pigeon, rhesus macaque, and garter snake. The three solitary species, octopus, panda, and Malayan sun bear, analyzed in this paper failed to demonstrate self-recognition. Several social animals also failed to demonstrate self-recognition, such as New Caledonian crows, the gray parrot, and sea lions, although this does not affect the comparison with solitary species. This hypothesis is strengthened by the social intelligence hypothesis, which suggests that social animals are more likely to boast greater cognitive abilities than solitary species due to more opportunities for cognitive challenge in complex social environments. Given the existing literature, this article proposes social animals are more likely to be self-aware than solitary ones.

## Data availability statement

The original contributions presented in the study are included in the article/Supplementary material, further inquiries can be directed to the corresponding author.

## Ethics statement

Ethical review and approval was not required for the animal study because this is a hypothesis article and all information is from open-access sources.

## Author contributions

The author confirms being the sole contributor of this work and has approved it for publication.

## Conflict of interest

The author declares that the research was conducted in the absence of any commercial or financial relationships that could be construed as a potential conflict of interest.

## Publisher’s note

All claims expressed in this article are solely those of the authors and do not necessarily represent those of their affiliated organizations, or those of the publisher, the editors and the reviewers. Any product that may be evaluated in this article, or claim that may be made by its manufacturer, is not guaranteed or endorsed by the publisher.
